# Supplementary material for: Oncomine™ Comprehensive Assay v3 vs. Oncomine™ Comprehensive Assay Plus
Source: Cancers (Basel). 2021 Oct 18;13(20):5230. doi: 10.3390/cancers13205230 (PMC8533843; doi:10.3390/cancers13205230)
Supplement: Supplementary file 1 [file cancers-13-05230-s001.zip › cancers-1392110-supplementary.pdf]

		OCAv3	OCA-Plus	Total
A)	**Initial number of unfiltered variants**	**n = 168,523**	**n = 374,230**	**n = 542,753**
**Overall filtering of variants**				
B)	**Gene & nucleotide-position filtering**			
	Variants found within overlapping genes of OCAv3^{M} & OCA-Plus	n = 161,810	n = 181,476	n = 343,286
	Variants found within overlapping nucleotide positions of OCAv3^{M} & OCA-Plus	n = 157,941	n = 162,456	n = 320,397
C)	**Pre-analysis data cleaning:**			
	Variants located within exonic region or in splise sites (-3/+3)	n = 147,165	n = 153,332	n = 300,497
	Variants being PASS by the Ion Reporter filter	n = 145,523	n = 141,085	n = 286,608
	Variants with nucleotide length of ≥ 1	n = 5,623	n = 5,213	n = 10,836
	Variants being SNV, MNV or indel	n = 5,623	n = 5,213	n = 10,836
D)	**Original variant filtering**			
	Variants absent of synonymous mutations	n = 3,393	n = 3,088	n = 6,481
	Variants not being a common SNP (UCSC common SNP)	n = 2,108	n = 1,877	n = 3,985
	Variants with an Ion Reporter p-value ≤ 0.01	n = 1,363	n = 1,862	n = 3,225
	Variants with a phred score ≥ 200	n = 855	n = 1,094	n = 1,949
	Variants with an allele ratio above first quartile	n = 693	n = 755	n = 1,448
	Variants not being potential germline	n = 395	n = 368	n = 763
	Variants with homopolymer content ≤ 4	n = 294	n = 275	n = 569
	Variants with a coverage above 10% of mean coverage	n = 235	n = 223	n = 458
	Variants with a coverage ≥ 100	n = 235	n = 223	n = 458
E)	**Benign/Germline mutations filtering**			
	Variants not being annotated in ClinVar as benign/germline	**n = 187**	**n = 183**	**n = 370**
**Rescue of variants**				
F)	**Below-Q1-variant filtering**	n = 162	n = 339	n = 501
	Variants passing cleaning	n = 28	n = 49	n = 77
	Variants passing specific criterions for filtering	**n = 6**	**n = 4**	**n = 10**
G)	**Low-base-coverage-variant filtering**	n = 59	n = 52	n = 111
	Variants passing cleaning	n = 12	n = 7	n = 19
	Variants passing specific criterions for filtering	**n = 3**	**n = 2**	**n = 5**
H)	**NOCALL-variant filtering**	n = 1,568	n = 12,380	n = 13,948
	Variants passing specific criterions for filtering	**n = 0**	**n = 1**	**n = 1**
I)	**Final variants**	**n = 196**	**n = 190**	**n = 386**

Figure S1: Overview of workflow data interpretation of 50 sample pairs used for sequencing with both OCAv3 and OCA-Plus.

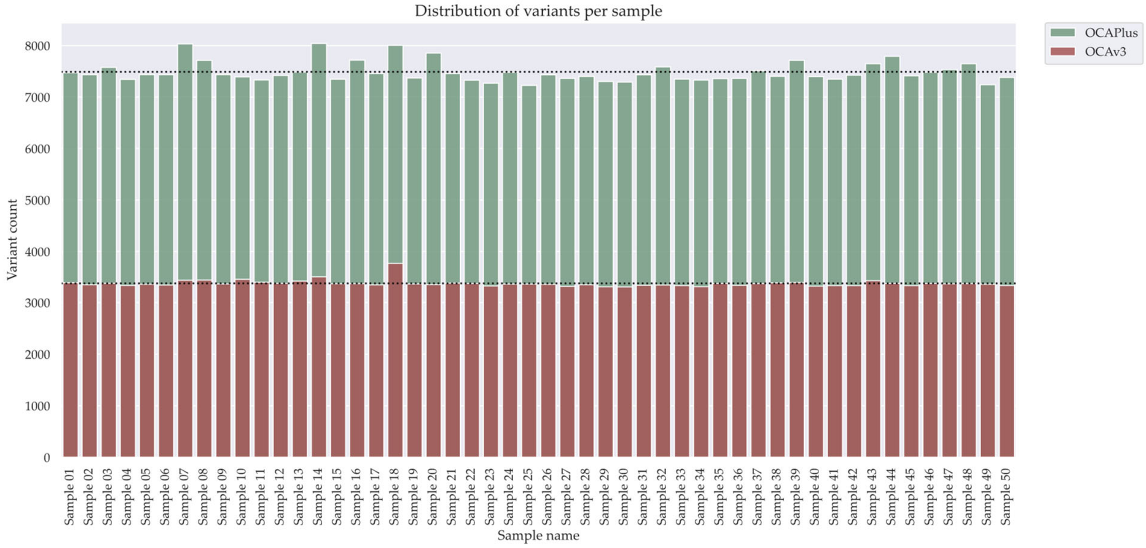

Figure S2: Distribution of total variants per sample of OCAv3 and OCA-Plus. Dotted horizontal lines represent average variant counts of 7489 and 3371 for OCA-Plus and OCAv3, respectively.

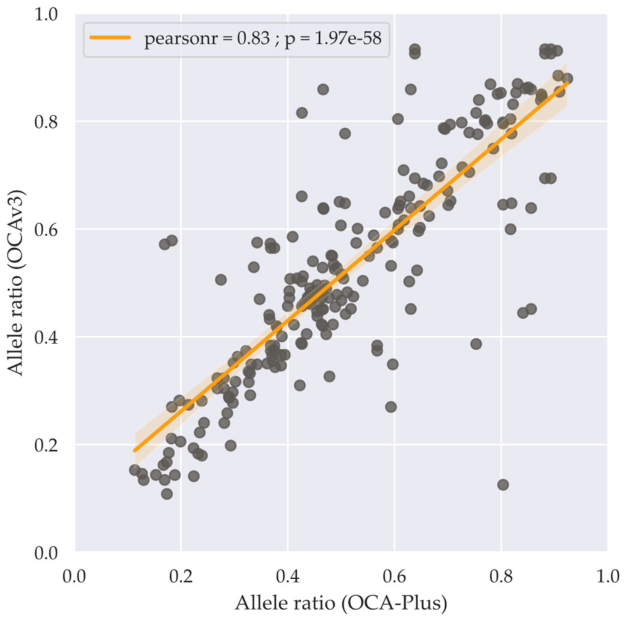

Figure S3: Scatter plot with Pearson correlation coefficient applied to allele ratios for variants identified in both OCA-Plus and OCAv3.

Table S1: Intersecting genes and unique genes of OCAv3 and OCA-Plus extracted from BED-files respectively. The following fusion genes for OCAv3 are not included in the list due to absence in the BED-file; *ERG, ETV1, ETV4, FGR, JAK2, MYB, MYBL1, NOTCH4, NRG1, NUTM1, PRKACA, PRKACB, RELA, RSPO2, RSPO3.*

OCAv3 unique genes (n=2)	OCA-Plus unique genes (n= 357)								Intersecting genes (n=144)		
CDK2	A1CF	CD163	ERAP1	KCNH7	OR2L2	**PPP2R2A**	SMAD2	ZIM3	AKT1	FLT3	**PALB2**
PPARG	ABCB1	CD274	ERAP2	KCNJ5	OR2L8	PPP6C	SMC1A	ZMYM3	AKT2	FOXL2	PDGFRA
	ABL1	CD276	ERCC4	KDM5C	OR2M3	PRDM1	SNCAIP	ZNF217	AKT3	GATA2	PDGFRB
	ABL2	CD79B	ERCC5	KDM6A	OR2T3	PRDM9	SOCS1	ZNF429	ALK	GNA11	PIK3CA
	ABRAXAS1	CDC73	ERRFI1	KEAP1	OR2T33	PRKACA	SOS1	ZNF479	AR	GNAQ	PIK3CB
	ACSM2B	CDH1	ETV6	KEL	OR2T4	PRKAR1A	SOX2	ZNF536	ARAF	GNAS	PIK3R1
	ACVR1	CDH10	FAM135B	KIR3DL1	OR2W3	PSMB10	SOX9	ZRSR2	ARID1A	H3F3A	PMS2
	ACVR1B	CDKN1A	FANCC	KLF4	OR4A15	PSMB8	SPEN		**ATM**	HIST1H3B	POLE
	ACVR2A	CDKN2C	FANCE	KLF5	OR4C15	PSMB9	SRSF2		ATR	HNF1A	PPP2R1A
	ADAM18	CHD4	FANCF	KLHL13	OR4C6	PTPRD	STAG2		ATRX	HRAS	PTCH1
	ADAMTS12	CIC	FANCG	KMT2A	OR4M1	PTPRT	STAT1		AXL	IDH1	PTEN
	ADAMTS2	CIITA	**FANCL**	KMT2B	OR4M2	PXDNL	STAT5B		BAP1	IDH2	PTPN11
	AMER1	CNTN6	FANCM	KMT2C	OR5D18	RAD52	STAT6		BRAF	IGF1R	RAC1
	ANO4	CNTNAP4	FAS	KMT2D	OR5F1	**RAD54L**	SUFU		**BRCA1**	JAK1	RAD50
	APC	CNTNAP5	FAT1	KRTAP2-1	OR5L1	RARA	SYT10		**BRCA2**	JAK2	RAD51
	ARHGAP35	COL11A1	FGF23	KRTAP6-2	OR5L2	RASA1	SYT16		BTK	JAK3	**RAD51B**
	ARID1B	CSMD3	FGF4	LARP4B	OR6F1	RASA2	TAF1		CBL	KDR	**RAD51C**
	ARID2	CTCF	FGF7	LATS1	OR8H2	RBM10	TAP1		CCND1	KIT	**RAD51D**
	ARID5B	CTLA4	FGF9	LATS2	OR8I2	RBP3	TAP2		CCND2	KNSTRN	RAF1
	ARMC4	CTNND2	FLT4	LRRC7	OR8U1	RECQL4	TAPBP		CCND3	KRAS	RB1
	ASXL1	CUL1	FOXA1	MAP2K7	ORC4	REG1A	TBX3		CCNE1	MAGOH	RET
	ASXL2	CUL3	FOXO1	MAP3K1	PAK5	REG1B	TCF7L2		**CDK12**	MAP2K1	RHEB
	ATP1A1	CUL4A	FUBP1	MAP3K4	PARP1	REG3A	TET2		CDK4	MAP2K2	RHOA
	AURKA	CUL4B	FYN	MAPK8	PARP2	REG3G	TGFBR1		CDK6	MAP2K4	RICTOR
	AURKB	CYLD	GALNT17	MARCO	PARP3	RGS7	TGFBR2		CDKN1B	MAPK1	RNF43
	AURKC	CYP2C9	GATA3	MCL1	PARP4	RIT1	TMEM132D		CDKN2A	MAX	ROS1
	AXIN1	CYP2D6	GLI1	MECOM	PAX5	RNASEH2A	TNFAIP3		CDKN2B	MDM2	SETD2
	AXIN2	CYSLTR2	GLI3	MEF2B	PBRM1	RNASEH2B	TNFRSF14		**CHEK1**	MDM4	SF3B1
	B2M	DAXX	GNA13	MEN1	PCBP1	RNASEH2C	TOP2A		**CHEK2**	MED12	SLX4
	**BARD1**	DCAF4L2	GPR158	MGA	PCDH17	RPA1	TP63		CREBBP	MET	SMAD4
	BCL2	DCDC1	GPS2	MITF	PDCD1	RPL10	TPMT		CSF1R	MLH1	SMARCA4
	BCL2L12	DDR1	GRID2	MLH3	PDCD1LG2	RPL22	TPP2		CTNNB1	MRE11	SMARCB1
	BCL6	DDX3X	H3F3B	MPL	PDE1A	RPL5	TPTE		DDR2	MSH2	SMO
	BCOR	DGCR8	HCN1	MSH3	PDE1C	RPS6KB1	TRHDE		EGFR	MSH6	SPOP
	BCR	DICER1	HDAC2	MTAP	PDIA3	RPTN	TRIM48		ERBB2	MTOR	SRC
	BLM	DNMT3A	HDAC9	MTUS2	PGD	RPTOR	TRIM51		ERBB3	MYC	STAT3
	BMP5	DOCK3	HIF1A	MUTYH	PHF6	RUNDC3B	TRRAP		ERBB4	MYCL	STK11
	BMPR2	DPYD	HIST1H1E	MYOD1	PIK3C2B	RUNX1	TSHR		ERCC2	MYCN	TERT
	BRINP3	DROSHA	HIST1H2BD	NCOR1	PIK3CD	RUNX1T1	UGT1A1		ESR1	MYD88	TOP1
	**BRIP1**	DSC1	HLA-A	NLRC5	PIK3CG	SDHA	USP8		EZH2	NBN	TP53
	C6	DSC3	HLA-B	NOL4	PIK3R2	SDHB	USP9X		FANCA	NF1	TSC1
	C8A	E2F1	HLA-C	NOTCH4	PIM1	SDHC	VHL		FANCD2	NF2	TSC2
	C8B	EIF1AX	ID3	NRXN1	PLCG1	SDHD	WAS		FANCI	NFE2L2	U2AF1
	CACNA1D	ELF3	IKBKB	NSD2	PLXDC2	SETBP1	WT1		FBXW7	NOTCH1	XPO1
	CALR	EMSY	IL6ST	NT5C2	PMS1	SH3RF2	XRCC2		FGF19	NOTCH2	
	CANX	ENO1	IL7R	NUP93	POLD1	SIX1	XRCC3		FGF3	NOTCH3	
	CARD11	EP300	INPP4B	NYAP2	POM121L12	SIX2	YAP1		FGFR1	NRAS	
	CASP8	EPAS1	IRF4	OR10G8	POT1	SLC15A2	YES1		FGFR2	NTRK1	
	CASR	EPCAM	IRS4	OR2G6	PPFIA2	SLC8A1	ZBTB20		FGFR3	NTRK2	
	CBFB	EPHA2	KCND2	OR2L13	PPM1D	SLCO1B3	ZFHX3		FGFR4	NTRK3	

Table S2: Tumor mutational burden (TMB) and Microsatellite instability (MSI) scores for samples 1–50.

Sample	TMB score	MSI Score	Sample	TMB score	MSI Score
Sample 01	8.5	0.0	Sample 26	5.7	2.8
Sample 02	9.5	2.9	Sample 27	3.8	3.3
Sample 03	14.2	14.7	Sample 28	4.8	9.4
Sample 04	1.9	2.9	Sample 29	0.0	4.8
Sample 05	3.8	4.0	Sample 30	1.0	4.6
Sample 06	3.8	1.9	Sample 31	1.9	3.8
Sample 07	11.4	2.8	Sample 32	5.7	2.5
Sample 08	11.6	15.3	Sample 33	4.7	2.0
Sample 09	1.9	2.7	Sample 34	2.9	13.1
Sample 10	14.3	4.9	Sample 35	5.7	4.8
Sample 11	4.8	0.7	Sample 36	1.9	16.5
Sample 12	2.8	0.6	Sample 37	3.8	2.0
Sample 13	8.6	1.3	Sample 38	5.7	2.9
Sample 14	16.2	0.7	Sample 39	10.5	0.7
Sample 15	3.8	0.9	Sample 40	2.9	3.9
Sample 16	6.6	0.8	Sample 41	2.9	2.2
Sample 17	6.6	8.2	Sample 42	2.9	1.1
Sample 18	18.1	2.2	Sample 43	18.9	9.5
Sample 19	2.8	6.3	Sample 44	2.9	3.8
Sample 20	1.9	1.5	Sample 45	4.7	6.2
Sample 21	7.6	5.8	Sample 46	9.5	0.7
Sample 22	0.0	0.6	Sample 47	3.8	5.4
Sample 23	1.0	4.8	Sample 48	10.4	1.0
Sample 24	1.9	2.7	Sample 49	25.3	2.2
Sample 25	1.0	2.0	Sample 50	4.8	3.1
